# Putative Modulation of OATP1A2 and P-gp Expression at the Blood–Brain Barrier by Nrf2–PXR: An Associative Hypothesis for Amyloid-β Transport in Alzheimer’s Disease

**DOI:** 10.3390/ijms27167264

**Published:** 2026-08-14

**Authors:** Lin Li, Yu Zhang, Sihong Li, Menghua Zhao, Weiqiang Hu, Yuwei Xiao, Jinhua Wen

**Affiliations:** 1 School of Pharmacy, Nanchang University, Nanchang 330006, China; llin202607@163.com (L.L.); yuzhang3319@163.com (Y.Z.); giaaaaaaaanna@163.com (S.L.); xiaoyuwei67@163.com (Y.X.); 2 Department of GCP, The First Affiliated Hospital of Nanchang University, Nanchang 330006, China; zhaomh699@163.com; 3 Department of Pharmacy, The First Affiliated Hospital of Nanchang University, Nanchang 330006, China; hwq199892@163.com; 4 Key Laboratory of New Drug Transformation and Evaluation of Jiangxi Province, Nanchang 330031, China

**Keywords:** Alzheimer’s disease, blood–brain barrier, Nrf2, PXR, OATP1A2, P-glycoprotein, crosstalk

## Abstract

Impaired amyloid-β (Aβ) clearance across the blood–brain barrier (BBB) is a major contributor to Aβ accumulation in Alzheimer’s disease (AD). P-glycoprotein (P-gp) has been identified as a key BBB efflux transporter involved in Aβ clearance, whereas emerging evidence suggests that organic anion transporting polypeptide 1A2 (OATP1A2) and its rodent counterparts, such as Oatp1a4, may participate in the influx component of Aβ transport. Nuclear factor erythroid 2–related factor 2 (Nrf2) and pregnane X receptor (PXR) are important transcriptional regulators of oxidative stress responses, xenobiotic metabolism, and transporter expression and may therefore modulate OATP1A2 and P-gp expression at the BBB. However, the mechanisms by which Nrf2–PXR crosstalk may regulate OATP1A2/P-gp expression in BBB endothelial cells under AD-relevant pathological conditions, as well as the consequences of this regulation for Aβ transport homeostasis, remain incompletely understood. This review summarizes current evidence linking P-gp, OATP1A2/Oatp1a4, Nrf2, and PXR to BBB transporter homeostasis in AD. A “net-effect” model is further proposed, in which Nrf2–PXR crosstalk may shift the BBB transporter balance toward enhanced P-gp-mediated efflux and reduced OATP1A2-associated influx. Because several key components of this model, particularly OATP1A2-mediated Aβ influx and BBB-specific Nrf2–PXR regulation, remain insufficiently validated, this model should be regarded as a mechanistic framework for future experimental investigation rather than as an established pathogenic pathway. Clarifying this regulatory axis may provide new insights into BBB dysfunction and impaired Aβ clearance in AD and may help identify potential molecular targets for therapeutic intervention.

## 1. Introduction

Alzheimer’s disease (AD) is a progressive neurodegenerative disorder affecting the central nervous system. Its major clinical manifestations include progressive memory impairment, cognitive decline, personality changes, language dysfunction, and other neuropsychiatric symptoms, which severely compromise patients’ quality of life and impose a substantial burden on families and society. With the acceleration of global population aging, the prevalence and socioeconomic burden of AD continue to increase, making it a major public health challenge worldwide [1]. The principal neuropathological hallmarks of AD include extracellular amyloid-β (Aβ) plaques, intracellular neurofibrillary tangles composed of hyperphosphorylated tau, neuronal loss, and synaptic degeneration [2]. Aβ accumulation has been proposed as an early contributor to AD pathogenesis; however, AD progression involves multiple interacting pathological processes, including tau pathology, neuroinflammation, oxidative stress, synaptic dysfunction, and vascular impairment [3]. Therefore, elucidating the mechanisms that regulate Aβ deposition and clearance is important for identifying potential therapeutic targets for AD.

Aberrant Aβ accumulation is a core pathological feature of AD, and impaired BBB-mediated clearance is considered an important mechanism contributing to cerebral Aβ deposition [4]. The BBB is a highly specialized interface between the blood and the brain parenchyma, comprising primarily brain microvascular endothelial cells, astrocytic end-feet, pericytes, basement membrane components, and associated supporting cells. By tightly regulating molecular exchange between the circulation and the brain, the BBB maintains cerebral microenvironmental homeostasis [5]. The structural integrity and physiological function of the BBB are essential for normal central nervous system function, whereas BBB dysfunction has been closely associated with the onset and progression of various neurological diseases, including AD [6].

Aβ homeostasis in the brain is governed by multiple clearance and transport pathways rather than by any single transporter system [7]. In addition to P-glycoprotein (P-gp)-mediated efflux [8], receptor-mediated transport mechanisms involving low-density lipoprotein receptor-related protein 1 (LRP1) and the receptor for advanced glycation end products (RAGE) contribute to bidirectional Aβ transport across the BBB [9,10]. LRP1 is primarily associated with Aβ efflux from the brain to the blood, whereas RAGE has been implicated in Aβ influx from the circulation into the brain [9,10]. Other ATP-binding cassette transporters, including breast cancer resistance protein/ATP-binding cassette subfamily G member 2 (BCRP/ABCG2), may also participate in BBB defense and Aβ transport regulation [11]. Moreover, perivascular drainage, cerebrospinal fluid turnover, and glymphatic clearance contribute to the removal of soluble Aβ from the brain interstitial compartment [7,12]. Therefore, the organic anion transporting polypeptide 1A2 (OATP1A2)/P-gp axis discussed in this review should be interpreted as a potential transporter-regulatory component within the broader Aβ clearance network, rather than as the sole mechanism responsible for Aβ accumulation in AD [7,8,13].

A variety of transport proteins are expressed at the BBB and play essential roles in regulating the exchange of endogenous metabolites, xenobiotics, and peptides between the blood and the brain. Among these transporters, OATP1A2 and P-gp are particularly relevant to BBB transport homeostasis [14,15]. OATP1A2 is encoded by the *SLCO1A2* gene. As a member of the organic anion transporting polypeptide family, OATP1A2 has been reported in brain- and BBB-related systems and has attracted increasing attention because of its broad substrate spectrum and potential relevance to drug entry into the central nervous system. OATP1A2 has been localized predominantly to the luminal membrane of brain microvascular endothelial cells, where it may mediate the transport of various endogenous compounds, including thyroid hormones and bile acids, as well as exogenous drugs, including statins and anticancer agents, from the blood into the brain [14]. Recent evidence further suggests that OATP1A2 may participate in Aβ_1–42_ uptake in experimental cell models [13]. However, whether OATP1A2 substantially contributes to Aβ influx across the human BBB in vivo remains unclear. Accordingly, OATP1A2 should currently be regarded as a potential participant in Aβ transport and processing rather than as an established determinant of cerebral Aβ accumulation [13,16].

**Figure 1:**
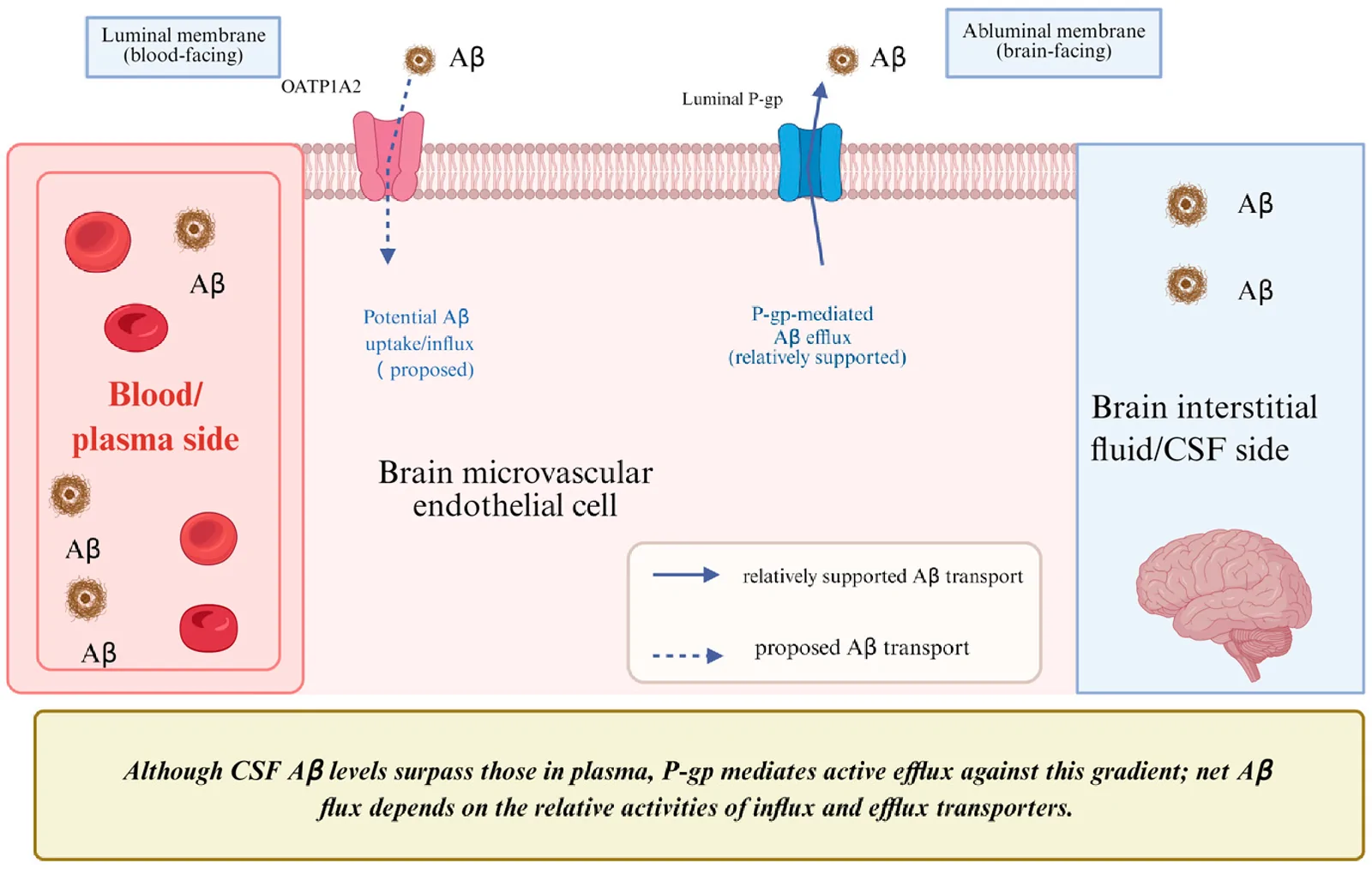
Proposed transporter balance governing amyloid-β (Aβ) flux across the blood–brain barrier (BBB). The schematic depicts brain microvascular endothelial cells separating the blood/plasma side from the brain interstitial fluid/cerebrospinal fluid (CSF) side. Organic anion transporting polypeptide 1A2 (OATP1A2) is presented as a proposed luminal route for Aβ uptake/influx, whereas luminal P-glycoprotein (P-gp) is presented as a relatively supported pathway for active Aβ efflux toward the blood. Solid arrows denote relatively supported transport pathways, whereas dashed arrows denote proposed or incompletely validated pathways. Although CSF Aβ levels may exceed plasma Aβ levels, active P-gp-mediated efflux suggests that net Aβ flux across the BBB may depend on transporter balance rather than passive diffusion alone. Created in BioRender. Zhang, Y. (2026) https://BioRender.com/u34x5e0.

P-gp, encoded by the *ABCB1* gene, is a member of the ATP-binding cassette (ABC) transporter family and is predominantly expressed on the luminal membrane of brain microvascular endothelial cells. As a major BBB efflux transporter, P-gp extrudes a wide range of metabolites, toxic compounds, and xenobiotics from the brain toward the circulation, thereby contributing to the maintenance of cerebral microenvironmental homeostasis [15]. A growing body of evidence indicates that P-gp plays an important role in Aβ clearance by facilitating Aβ efflux from the brain and reducing cerebral Aβ deposition [17]. Reduced P-gp expression or activity may impair BBB-mediated Aβ clearance, thereby contributing to Aβ accumulation and AD-related pathology [18]. Therefore, Aβ transport across the BBB may be influenced by the balance between efflux transporters such as P-gp and uptake transporters such as OATP1A2 [13,16,17]. Under AD-relevant pathological conditions, reduced P-gp-mediated efflux, together with potential alterations in OATP1A2-mediated uptake, may disrupt BBB Aβ transport homeostasis and contribute to cerebral Aβ accumulation [13,16,17,18]. Thus, the coordinated regulation of OATP1A2 and P-gp at the BBB may represent an important component of Aβ transport homeostasis, as illustrated in Figure 1.

As a key protective interface of the central nervous system (CNS), the BBB relies on multiple transporters to maintain selective permeability and regulate molecular exchange between the blood and the brain. Nuclear factor erythroid 2–related factor 2 (Nrf2) and pregnane X receptor (PXR) are intracellular transcriptional regulators that may influence BBB transporter expression through nuclear transcriptional mechanisms. Through modulation of transporters involved in xenobiotic efflux, metabolic defense, and barrier protection, these transcriptional pathways contribute to CNS homeostasis and responses to exogenous insults and pathological stress [19].

Nrf2 is a central regulator of the cellular antioxidant response. Under basal conditions, Nrf2 is sequestered in the cytoplasm by its association with Kelch-like ECH-associated protein 1 (Keap1), which promotes Nrf2 degradation. In response to oxidative stress, electrophiles, or other activating stimuli, Nrf2 is released from Keap1-mediated repression, translocates into the nucleus, binds to antioxidant response elements (AREs), and initiates the transcription of downstream cytoprotective genes [20]. At the BBB, Nrf2 has been implicated in the regulation of several transporters, particularly members of the ATP-binding cassette (ABC) transporter family. Nrf2 activation has been reported to increase the expression and/or transport activity of P-gp (*ABCB1*), BCRP (*ABCG2*), and multidrug resistance-associated protein 2 (MRP2; *ABCC2*), thereby enhancing BBB detoxification capacity [21]. In addition, Nrf2 has been reported to possess epigenetic regulatory functions through the modulation of histone deacetylases (HDACs), DNA methyltransferases (DNMTs), and microRNA (miRNA) biogenesis [22]. In parallel, DNA methylation and histone modifications have been implicated in the epigenetic regulation of selected solute carrier organic anion transporter (*SLCO*)/OATP family members, such as *SLCO1B3*/OATP1B3, in cancer cell models [23]. Taken together, these findings raise the possibility that stress-responsive transcriptional programs may indirectly influence OATP-related transporter expression through epigenetic mechanisms. However, direct evidence demonstrating Nrf2-mediated regulation of *SLCO1A2*/OATP1A2 in human BBB endothelial cells is currently lacking.

PXR is a ligand-activated nuclear receptor that has been implicated in xenobiotic metabolism and detoxification, including responses to therapeutic drugs and environmental chemicals. A broad range of xenobiotics and endogenous compounds, including rifampicin and steroid-related molecules, have been identified as PXR ligands [24]. Upon activation, PXR forms a heterodimer with retinoid X receptor (RXR), binds to PXR response elements (PXREs) in the promoter regions of target genes, and regulates downstream gene transcription [25]. At the BBB, PXR activation has been reported to regulate BBB efflux transporters, particularly P-gp [26]. By contrast, although PXR-mediated regulation of OATP1A2 expression has been demonstrated in non-BBB cellular contexts, direct evidence supporting PXR-mediated regulation of OATP1A2 at the human BBB remains limited [27].

**Figure 2:**
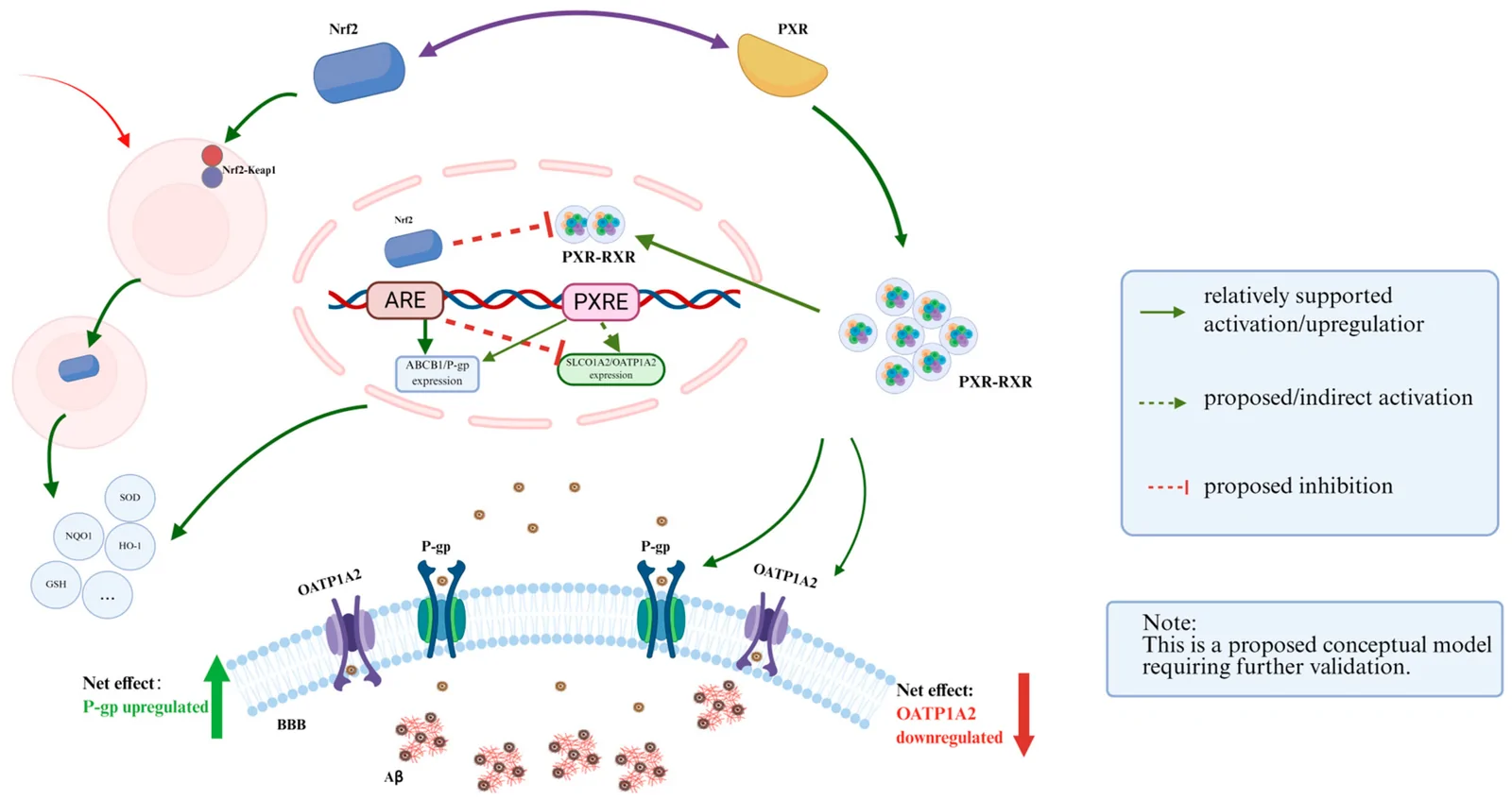
Proposed nuclear factor erythroid 2–related factor 2 (Nrf2)–pregnane X receptor (PXR) crosstalk model linking BBB transporter regulation to Aβ transport homeostasis. The schematic summarizes a hypothesis-generating framework in which intracellular Nrf2 and PXR signaling may regulate BBB transporter expression through nuclear transcriptional mechanisms. Nrf2 activation is proposed to support *ABCB1*/P-gp expression and antioxidant responses, whereas PXR may regulate transporter gene expression after heterodimerization with retinoid X receptor (RXR) [21,26,28,29]. Potential Nrf2–PXR crosstalk may further influence transporter balance through indirect epigenetic or PXR/RXR-related mechanisms [22,23,27,30,34]. Therefore, the proposed net effect should be interpreted as a possible shift toward enhanced P-gp-associated efflux and potentially reduced OATP1A2-associated uptake/influx, rather than as an established BBB-specific pathway. Solid green arrows denote relatively supported activation/upregulation pathways, dashed green arrows denote proposed or indirect activation pathways, and dashed red blunt-ended lines denote proposed inhibitory pathways. This model should be regarded as a conceptual framework requiring further validation in BBB-specific and Alzheimer’s disease (AD)-relevant experimental systems. **Abbreviations:** Keap1, Kelch-like ECH-associated protein 1; ARE, antioxidant response element; PXRE, PXR response element; NQO1, NAD(P)H quinone oxidoreductase 1; HO-1, heme oxygenase-1; SOD, superoxide dismutase; GSH, glutathione. Created in BioRender. Zhang, Y. (2026) https://BioRender.com/u34x5e0.

In summary, the available literature suggests that both Nrf2 and PXR may influence OATP1A2 and P-gp expression, although the extent of direct regulation appears to differ among transporters. These pathways may interact in ways that affect BBB transporter function; however, such interactions remain incompletely validated, particularly under BBB-specific and AD-relevant conditions (Figure 2). Potential Nrf2–PXR crosstalk has been proposed in several cellular contexts; however, its functional relevance at the BBB remains insufficiently defined. With respect to the P-gp-related arm, Nrf2 activation may directly enhance P-gp expression and/or transport activity, whereas PXR signaling may also support P-gp transcriptional regulation [21,26,28,29]. Although Nrf2–PXR crosstalk could, under certain conditions, attenuate PXR-dependent support for P-gp through coactivator- or RXR-related mechanisms [30,31,32,33,34], this indirect regulatory route may operate over a longer time scale than direct ARE- or PXRE-mediated transcriptional activation and remains to be validated in BBB-specific systems.

By contrast, the evidence supporting Nrf2/PXR-mediated regulation of OATP1A2 is more complex. To date, direct evidence demonstrating that either Nrf2 or PXR regulates *SLCO1A2*/OATP1A2 expression in human BBB endothelial cells is still lacking. Therefore, in this review, the proposed Nrf2–PXR–OATP1A2 relationship is interpreted as an indirect regulatory hypothesis based on non-BBB evidence and related mechanistic inference, rather than as an established BBB-specific pathway. A previous study has shown that *Schisandra chinensis* extract (SCE) activates Nrf2 signaling and promotes Nrf2 nuclear translocation in HepG2 cells, accompanied by increased P-gp expression and decreased OATP1A2 expression. Moreover, *NFE2L2* small interfering RNA (siRNA) knockdown attenuated the regulatory effects of SCE on related transporters, suggesting that Nrf2-mediated signaling may participate in the regulation of P-gp/OATP1A2 transporter balance [35]. In addition, Nrf2 activators have been reported to alter the expression of selected hepatic mouse Oatp transporters [36], and OATP1A2 itself has been shown to undergo epigenetic regulation through the histone deacetylase 6 (HDAC6)–general control nonderepressible 5 (GCN5)/ E1A-binding protein p300 (p300)/CREB-binding protein (CBP)-associated factor (PCAF)–histone H3 lysine 9 acetylation (H3K9Ac) axis in non-BBB cancer models [37]. Compared with the indirect evidence linking Nrf2 to OATP1A2 regulation, the evidence supporting PXR-mediated regulation of OATP1A2 is relatively more direct. Rifampicin-activated PXR has been reported to induce OATP1A2 expression in breast cancer cell models, and a PXR response element in the OATP1A2 promoter supports PXR-dependent transcriptional regulation [27]. Based on these findings, Nrf2 may indirectly influence OATP1A2 expression through epigenetic regulatory networks and potential attenuation of PXR-dependent transcriptional support (Figure 2). However, whether this proposed Nrf2–PXR–OATP1A2 regulatory chain operates in human BBB endothelial cells remains to be validated in BBB-specific and AD-relevant experimental systems.

Taken together, BBB transporter function may be regulated by Nrf2 activation through a multilayered regulatory network that remains incompletely validated. The P-gp-related arm is supported by comparatively stronger evidence, as Nrf2-mediated transcriptional regulation of ABC transporters, including P-gp, may enhance efflux capacity and thereby facilitate Aβ clearance [21,38]. By contrast, support for the OATP1A2-related arm remains more indirect, because the proposed regulation of OATP1A2 by Nrf2/PXR is mainly inferred from non-BBB cellular models, epigenetic regulatory mechanisms, hepatic Oatp transporter studies, and promoter-level evidence [27,35,36,37]. Therefore, the proposed dual regulatory pattern—enhanced P-gp-associated efflux together with potentially reduced OATP1A2-associated uptake—should be interpreted as a literature-supported, testable hypothesis rather than as an established BBB-specific mechanism. This framework raises an important scientific question: whether an Nrf2-mediated shift in transporter balance toward increased efflux and reduced uptake may represent an endogenous protective response that limits Aβ accumulation under AD-relevant pathological conditions. On the basis of this hypothesis, this review evaluates the potential role of the Nrf2–PXR regulatory axis in modulating BBB transporter function and discusses the prospects and challenges of targeting this signaling network as a potential strategy for ameliorating BBB dysfunction in AD.

To enhance the rigor of this narrative review while minimizing overinterpretation, the relevant evidence was critically assessed according to its biological context and mechanistic relevance to the proposed Nrf2–PXR–OATP1A2/P-gp axis. Priority was given to studies involving BBB models, human brain tissue, transporter function, transcriptional regulation, and AD-relevant pathological conditions. Because some mechanistic links are derived from animal models, non-BBB tissues, or promoter-level inference, they should not be interpreted as established BBB-specific mechanisms. Accordingly, these lines of evidence were integrated to develop a conceptual and testable framework for future validation, rather than being presented as a confirmed BBB-specific regulatory mechanism.

## 2. Recent Advances in Nrf2 Research and Transporter-Mediated Regulatory Mechanisms

Nrf2 is a central transcription factor that regulates cellular redox homeostasis and may influence BBB transporter expression. Under basal conditions, Nrf2 is sequestered in the cytoplasm by Keap1, which functions as a substrate adaptor for the Cullin 3 (Cul3)-based E3 ubiquitin ligase complex. This interaction promotes Nrf2 ubiquitination and subsequent proteasomal degradation, thereby maintaining low basal Nrf2 activity. In addition to this canonical Keap1-dependent pathway, signaling cascades such as the phosphoinositide 3-kinase/protein kinase B (PI3K/Akt) pathway may modulate Nrf2 activity through phosphorylation-dependent mechanisms [39]. Upon oxidative stress or electrophilic stimulation, critical cysteine residues in Keap1 are modified, thereby weakening Keap1-mediated Nrf2 ubiquitination and allowing Nrf2 stabilization, nuclear translocation, and transcriptional activation. In the nucleus, Nrf2 binds to AREs and induces the coordinated expression of cytoprotective genes, including those encoding NAD(P)H quinone oxidoreductase 1 (NQO1), heme oxygenase-1 (HO-1), superoxide dismutases (SODs), and enzymes involved in glutathione (GSH) synthesis and recycling [40].

During the pathological progression of AD, dysregulation of the Nrf2–ARE pathway may contribute to BBB dysfunction, oxidative injury, and neuronal damage. Activation of this pathway may attenuate Aβ-associated pathology by enhancing antioxidant and detoxification responses, and has therefore been investigated as a potential therapeutic strategy for AD [41]. Several studies have reported reduced Nrf2 expression or impaired Nrf2 activity in brains from patients with AD and in experimental models. In AD models, Nrf2 activators such as sulforaphane (SFN) have been reported to activate Nrf2 signaling and attenuate abnormal Aβ accumulation [42]. Notably, Nrf2 may also participate in epigenetic regulation by modulating DNMTs, HDACs, and miRNA biogenesis [22,43]. Because promoter methylation and histone modifications have been implicated in the regulation of selected *SLCO*/OATP family members, such as *SLCO1B3*/OATP1B3, in cancer cell models [23], Nrf2-related epigenetic regulation may represent a potential indirect route for modulating transporter-related gene expression. However, direct evidence linking Nrf2 activity to *SLCO1A2*/OATP1A2 regulation in human BBB endothelial cells is currently lacking, and its impact on Aβ flux across the BBB remains to be experimentally validated. Overall, Nrf2 activation reduces reactive oxygen species (ROS) levels through antioxidant pathways and provides a rationale for future studies evaluating its role in preserving proteostasis, epigenetic homeostasis, and BBB transporter function in AD-relevant contexts [44].

## 3. Recent Advances in PXR Signaling and Transporter-Mediated Regulation

PXR is a member of nuclear receptor subfamily 1 group I (NR1I) within the nuclear receptor superfamily and is highly expressed in the liver and intestine, with additional expression reported in brain microvascular endothelial cells (BMECs) at the BBB. Structurally, PXR contains a conserved DNA-binding domain (DBD) and a ligand-binding domain (LBD) with broad ligand-binding capacity. The large and flexible hydrophobic pocket within the LBD enables PXR to recognize a wide range of endogenous and exogenous ligands with diverse chemical structures [45]. As a ligand-activated xenobiotic sensor, PXR plays a central role in xenobiotic clearance, drug metabolism, and the maintenance of endogenous metabolic homeostasis [24].

Upon activation by steroid hormones, bile acids, clinical drugs such as rifampicin, or environmental chemicals, PXR undergoes conformational changes and forms a heterodimer with RXR. This heterodimer binds to PXR response elements (PXREs) in the promoter regions of target genes and recruits transcriptional coactivators, thereby inducing the expression of genes involved in drug metabolism and transport, including cytochrome P450 family 3 subfamily A member 4 (CYP3A4) and cytochrome P450 family 2 subfamily B member 6 (CYP2B6), as well as P-gp [25,46]. In addition to efflux transporters, PXR has also been implicated in the regulation of selected OATP isoforms. Functional PXREs have been identified in the upstream regulatory regions of *SLCO1B1* and *SLCO1B3*, which encode OATP1B1 and OATP1B3, respectively. These transporters are predominantly expressed in the liver and have been reported to be regulated by PXR activation [27,47]. However, these findings should not be directly extrapolated to OATP1A2 regulation at the human BBB without further validation.

Within the BBB defense system, PXR may contribute to the regulation of polarized expression and function of transporters. In this context, P-gp is one of the best-characterized PXR-responsive transporters. By binding to response elements within the *ABCB1* promoter region, activated PXR can upregulate P-gp expression, thereby enhancing efflux transport capacity [28,48]. Because P-gp is a major xenobiotic efflux transporter at the BBB, PXR-mediated upregulation of P-gp may enhance BBB efflux capacity and limit the brain accumulation of selected xenobiotics and endogenous substrates [29]. Experimental studies suggest that PXR activation may restore or enhance P-gp expression and transport function at the compromised BBB, thereby facilitating Aβ efflux and reducing cerebral Aβ burden in AD models [49]. In addition, Aβ itself has been reported to affect ABC transporter expression and activity in brain endothelial models [50]. Nevertheless, whether this mechanism operates to a similar extent in the human BBB in AD remains to be established.

Overall, PXR signaling represents a potentially important regulatory pathway linking xenobiotic sensing, BBB transporter regulation, and Aβ transport homeostasis. However, in the complex pathological environment of AD, whether altered PXR expression involves promoter-level epigenetic regulation, and how PXR interacts with other stress-responsive pathways such as Nrf2, remain important questions requiring further investigation.

## 4. Recent Advances in OATP1A2 and P-gp at the BBB

As a critical interface between the blood and the brain, the BBB plays an essential role in maintaining CNS homeostasis and protecting neural tissue from potentially harmful substances [51]. OATP1A2 and P-gp have both been reported to be localized at the luminal membrane of BMECs, where they may contribute to the polarized transport architecture of the BBB [52,53]. Functionally, OATP1A2 is generally associated with substrate uptake from the blood-facing side, whereas P-gp mediates ATP-dependent efflux from endothelial cells toward the circulation. Thus, these transporters may influence the net movement of selected substrates across the BBB through opposing but functionally coordinated transport processes.

OATP1A2 is a member of the OATP family and is encoded by the *SLCO1A2* gene. It mediates the uptake of a broad range of endogenous compounds, including thyroid hormones and bile acids, as well as exogenous drugs, from the blood into cells. Impaired OATP1A2 function may alter CNS exposure to selected substrates and thereby influence neurochemical homeostasis [13,54]. In the context of AD, however, the role of OATP1A2 in Aβ transport remains incompletely established. Recent evidence suggests that OATP1A2 may participate in Aβ_1–42_ uptake in experimental cell models [13]. For example, OATP1A2-overexpressing cells have been reported to exhibit increased Aβ_1–42_ uptake compared with control cells, suggesting that OATP1A2 may function as a potential Aβ uptake transporter under specific experimental conditions [13]. Nevertheless, whether OATP1A2 substantially contributes to Aβ influx across the human BBB in vivo remains to be determined.

By contrast, P-gp is a well-characterized BBB efflux transporter involved in the clearance of xenobiotics, toxic metabolites, and Aβ-related substrates from the brain toward the circulation. Reduced P-gp activity in AD may result from decreased transporter abundance, impaired efflux function, or both. Positron emission tomography (PET) studies have reported reduced BBB P-gp function in patients with AD, including those with mild AD [55,56]. Mechanistically, Aβ_40_ has been shown in experimental systems to induce P-gp ubiquitination, internalization, and proteasome-dependent degradation, thereby reducing P-gp protein expression and transport activity [15]. However, whether reduced P-gp activity is directly associated with Braak staging of neurofibrillary tangles remains unclear. Although local P-gp loss has been linked to neurofibrillary tangle and amyloid plaque burden [57], quantitative targeted proteomic analysis found no significant effect of Braak stage on overall P-gp abundance at the human BBB [58]. Therefore, current evidence supports an association between P-gp impairment and local AD-related lesion burden, but does not establish a direct monotonic relationship between P-gp transport activity and Braak stage.

The co-localization of P-gp and OATP1A2 at the BBB luminal membrane, together with their opposing orientations in substrate uptake and efflux, suggests that these transporters should not be considered in isolation. Instead, their coordinated regulation may contribute to determining the net flux of substrates across the BBB [59]. Transporter expression and function are controlled by complex signaling networks, among which Nrf2 is an important stress-responsive transcriptional regulator. Under oxidative stress, Nrf2 activation may enhance cellular antioxidant and metabolic defense. However, prolonged pathological stress may also engage epigenetic mechanisms, including DNMT-mediated DNA methylation, potentially leading to altered transcription of selected transporter-related genes and remodeling of transporter expression [60]. Importantly, such mechanisms remain incompletely validated for *SLCO1A2*/OATP1A2 regulation in human BBB endothelial cells. Beyond AD, transporter dysregulation has also been implicated in other neurological disorders, including Parkinson’s disease and epilepsy, where changes in P-gp and other BBB efflux transporter functions may contribute to altered drug responsiveness and disease progression [61]. These observations further support the importance of transporter balance at the BBB and emphasize the need for disease- and tissue-specific validation.

## 5. Molecular Logic and Tissue Specificity of Nrf2–PXR Signaling Crosstalk

As key transcriptional regulators of cellular stress responses and metabolism, Nrf2 and PXR may interact through complex and context-dependent mechanisms that contribute to cellular homeostasis [62,63,64]. Under basal physiological conditions, Nrf2 is maintained at a low level through Keap1-dependent ubiquitination and proteasomal degradation, thereby preserving a sensitive threshold for oxidative stress responses [65]. In parallel, PXR may support detoxification capacity through ligand-dependent activation and heterodimerization with retinoid X receptor alpha (RXRα), thereby regulating the expression of selected detoxification enzymes and transporters [66,67,68]. In this basal state, Nrf2- and PXR-regulated pathways may function in a complementary manner: Nrf2 helps maintain GSH-dependent antioxidant capacity, whereas PXR-regulated metabolic enzymes and transporters contribute to xenobiotic metabolism and efflux. Together, these pathways may support basal cellular detoxification and defense capacity [64,69,70].

However, under severe oxidative stress or high xenobiotic exposure, Nrf2 and PXR may shift from complementary cooperation to functional competition. One proposed mechanism involves competition for shared transcriptional coactivators, particularly CBP/p300, which links nuclear receptors and stress-responsive transcription factors to the basal transcriptional machinery [30,31,63,71,72]. The availability of free CBP/p300 is limited, and this stoichiometric constraint may contribute to transcriptional interference between Nrf2- and PXR-dependent pathways. Biophysical and molecular studies suggest that the Nrf2-ECH homology 4/5 (Neh4/5) transactivation domains of Nrf2 may recruit CBP/p300, whereas activated PXR–RXRα complexes may also require CBP/p300 for transcriptional activation [32]. Therefore, strong activation of PXR by potent ligands such as rifampicin may alter the recruitment or availability of transcriptional coactivators involved in xenobiotic-response gene regulation, although direct evidence that this limits Nrf2-dependent transcription through CBP/p300 competition remains insufficient [33,73]. This potential coactivator-based crosstalk model is consistent with observations that PXR-regulated xenobiotic-response pathways and electrophile-responsive/Nrf2-related gene programs may be differentially modulated under chemical or dietary stimulation [74].

In addition to coactivator competition, direct protein–protein interactions may also contribute to Nrf2–PXR crosstalk. The Nrf2-ECH homology 7 (Neh7) domain of Nrf2 has been implicated in negative regulation mediated by nuclear receptors. For example, RXRα may interact with the Neh7 domain and interfere with Nrf2 transactivation by limiting cofactor recruitment [34,75,76]. This observation suggests that RXRα-containing nuclear receptor complexes may suppress Nrf2 activity under specific conditions. Conversely, Nrf2 may also influence PXR signaling. Studies showing that hepatic PXR expression is reduced in Nrf2-deficient mice suggest that Nrf2 may contribute to the maintenance of basal PXR expression [77]. However, whether activated Nrf2 directly suppresses PXR transcriptional activity through coactivator competition or protein–protein interactions remains incompletely established. Thus, the Nrf2–PXR relationship should be interpreted as a context-dependent regulatory network rather than a fixed synergistic or antagonistic pathway.

The interplay between Nrf2 and PXR also exhibits marked tissue specificity. In the liver, where PXR is highly expressed, PXR plays a dominant role in xenobiotic metabolism and may negatively regulate selected Nrf2-dependent antioxidant pathways [63,78]. Upon activation by xenobiotics, PXR has been reported to suppress the expression of certain Nrf2-regulated phase II enzymes, such as NQO1, thereby reshaping the balance between oxidative stress defense and cytochrome P450 (CYP)-mediated metabolism [79]. In liver injury contexts, altered PXR and Nrf2 signaling may reshape the balance between xenobiotic metabolism and antioxidant defense, potentially influencing hepatocyte stress responses, including ferroptosis or apoptosis [80,81,82].

By contrast, in the kidney, an excretory organ with high metabolic demand, Nrf2 and PXR may exhibit a more coordinated protective relationship. In hyperuricemia-related contexts, PXR activation has been reported to reduce lysine 48 (K48)-linked ubiquitination of Nrf2 and promote phosphorylation of Nrf2 at serine 40 (S40), thereby activating Nrf2 signaling and upregulating downstream cytoprotective genes [83]. In addition, ABCG2, an important urate transporter, may be transcriptionally regulated by both PXR and Nrf2. This dual regulation may allow the kidney to enhance urate excretion while simultaneously strengthening antioxidant defense [84,85].

Taken together, available evidence indicates that Nrf2–PXR crosstalk is highly tissue- and context-dependent. In the liver, PXR-dominant signaling may antagonize selected Nrf2-mediated antioxidant responses to preserve xenobiotic metabolic specificity, whereas in the kidney, Nrf2 and PXR may cooperate to support excretory and antioxidant functions under metabolic stress. This tissue-specific signaling logic provides an important framework for understanding organ-specific stress adaptation. However, whether similar patterns of Nrf2–PXR interaction occur at the BBB, particularly under AD-relevant pathological conditions, remains to be directly validated.

## 6. Concluding Hypothesis: The “Net-Effect” Model of the Nrf2–PXR Axis

Cerebral Aβ deposition is closely associated with impaired BBB-mediated clearance, with transporter systems such as OATP1A2 and P-gp potentially playing important but distinct roles. Under AD-relevant pathological conditions, the expression and function of BBB transporters may undergo substantial alterations. In animal models of AD, P-gp messenger RNA (mRNA) and protein levels, as well as P-gp transport activity, have been reported to decline [86,87]. This association has been further supported by analyses of human brain tissue, which showed that P-gp protein density in the temporal cortex of patients with AD was reduced compared with that in healthy controls [88]. In parallel, reduced mRNA and protein expression of *Slco1a4*/Oatp1a4, a rodent counterpart of human OATP1A2, has been reported in the brains of amyloid precursor protein/presenilin 1 (APP/PS1) mice [89]. These findings suggest that BBB transporter networks involved in Aβ handling are altered in AD, although the consequences of OATP-related changes for net Aβ influx remain incompletely defined.

As upstream regulators of transporter expression and cellular defense responses, Nrf2 and PXR signaling pathways may also be altered during AD progression, including impaired Nrf2 activity and reduced PXR expression reported in certain experimental contexts [42,50]. In AD-relevant oxidative stress contexts, Aβ-associated Akt/glycogen synthase kinase 3 beta (GSK-3β)/Fyn signaling may reduce nuclear Nrf2 availability and downstream antioxidant responses, thereby potentially weakening Nrf2-dependent BBB transporter regulation [21,90,91]. In addition, reduced Nrf2 activity may be associated with epigenetic repression of *NFE2L2*, the gene encoding Nrf2, including DNMT-mediated promoter hypermethylation. Such epigenetic repression may contribute to persistent impairment of antioxidant defense, although its consequences for BBB transporter-regulatory capacity remain to be directly validated [22,92]. Notably, PXR activation can upregulate P-gp expression, and restoration of P-gp activity may facilitate Aβ clearance; however, PXR activation may also influence uptake transporters in a context-dependent manner [26,27,28,29,93]. Whether PXR activation promotes OATP1A2 expression at the human BBB, and whether such an effect would increase Aβ influx at specific pathological stages, remain to be directly validated [27].

Based on these observations, Nrf2 has been implicated as a potential regulatory node that may influence BBB transport homeostasis. Future studies may evaluate whether enhancing P-gp-mediated efflux while limiting OATP1A2-associated uptake could theoretically shift the BBB transport balance toward Aβ clearance. However, this possibility should not be interpreted as an established intervention, because the ability of Nrf2 activation to suppress OATP1A2 expression at the human BBB remains hypothetical. Moreover, although Nrf2–PXR/RXR-related crosstalk has been reported in non-BBB contexts [30,31,32,33,34], whether Nrf2 competitively attenuates PXR-dependent transcriptional activity in BBB endothelial cells remains hypothetical and requires further experimental confirmation. Therefore, elucidating how the Nrf2–PXR axis regulates the P-gp/OATP1A2 balance is central to understanding BBB functional remodeling during AD progression and to exploring strategies to enhance Aβ clearance.

With respect to the P-gp-related arm, both Nrf2 and PXR may contribute to P-gp upregulation at the BBB. Nrf2 activation may directly enhance P-gp expression and/or transport activity through ARE-dependent stress-responsive transcriptional mechanisms [21,94,95], whereas PXR activation may also promote P-gp expression through PXR-dependent transcriptional regulation [26,28,29]. However, as discussed in Section 5, Nrf2–PXR crosstalk may involve competition for shared transcriptional coactivators or RXR-related regulatory interactions, raising the possibility that strong Nrf2 activation could indirectly attenuate PXR-dependent transcriptional support for P-gp under certain conditions [30,31,32,33,34]. Thus, Nrf2 activation may exert two opposing influences on P-gp regulation: a direct positive effect mediated by Nrf2-dependent transcriptional activation and a proposed indirect negative effect mediated by potential attenuation of PXR-related transcriptional support. Because the latter pathway is indirect, context-dependent, and not yet directly validated in BBB endothelial models, it is hypothesized here that, under AD-relevant oxidative stress conditions, the direct Nrf2-mediated activation of P-gp may predominate, leading to a net increase in P-gp expression and/or transport activity. Nevertheless, this proposed net effect remains to be validated in BBB endothelial models and AD-relevant experimental systems.

Compared with the P-gp-related arm, support for the OATP1A2-related arm remains more indirect and less fully validated. Current support for Nrf2/PXR-related regulation of OATP1A2 is derived primarily from non-BBB cellular models, hepatic Oatp transporter studies, epigenetic regulatory mechanisms, and promoter-level evidence for PXR-dependent transcriptional regulation of OATP1A2 [27,35,36,37]. Thus, Nrf2 activation may indirectly influence OATP1A2-associated uptake capacity through epigenetic regulatory networks and potential attenuation of PXR-dependent transcriptional support. However, direct evidence demonstrating that this proposed Nrf2–PXR–OATP1A2 regulatory chain operates in human BBB endothelial cells is still lacking, and whether it produces a measurable net effect on BBB Aβ transport remains hypothetical.

In summary, this review proposes a conceptual “net-effect” model of the Nrf2–PXR axis to integrate these complex and sometimes divergent regulatory pathways (Figure 2). In this hypothetical framework, Nrf2 is proposed as a potential regulatory hub for modulating BBB transport homeostasis. Under AD-relevant pathological conditions, Nrf2 activation may produce a net effect characterized by enhanced P-gp-mediated efflux and reduced OATP1A2-associated uptake, thereby shifting the Aβ transport balance toward clearance rather than accumulation. This predicted net effect is based on the differential regulation of P-gp and OATP1A2: for P-gp, direct Nrf2-mediated transcriptional upregulation could predominate over any indirect inhibitory influence mediated through PXR signaling; for OATP1A2, Nrf2 may theoretically reduce transporter expression through DNMT-related epigenetic mechanisms and attenuation of PXR-dependent transcriptional support. This model integrates the potential differential regulation of P-gp and OATP1A2 by Nrf2–PXR crosstalk and provides a conceptual framework for investigating BBB functional remodeling during AD progression. However, the specific predictions of this model, including net P-gp upregulation and OATP1A2 downregulation, remain to be experimentally validated. Therefore, the proposed net-effect model should currently be interpreted as a testable hypothesis rather than as a confirmed mechanism.

## 7. Conclusions

**Figure 3:**
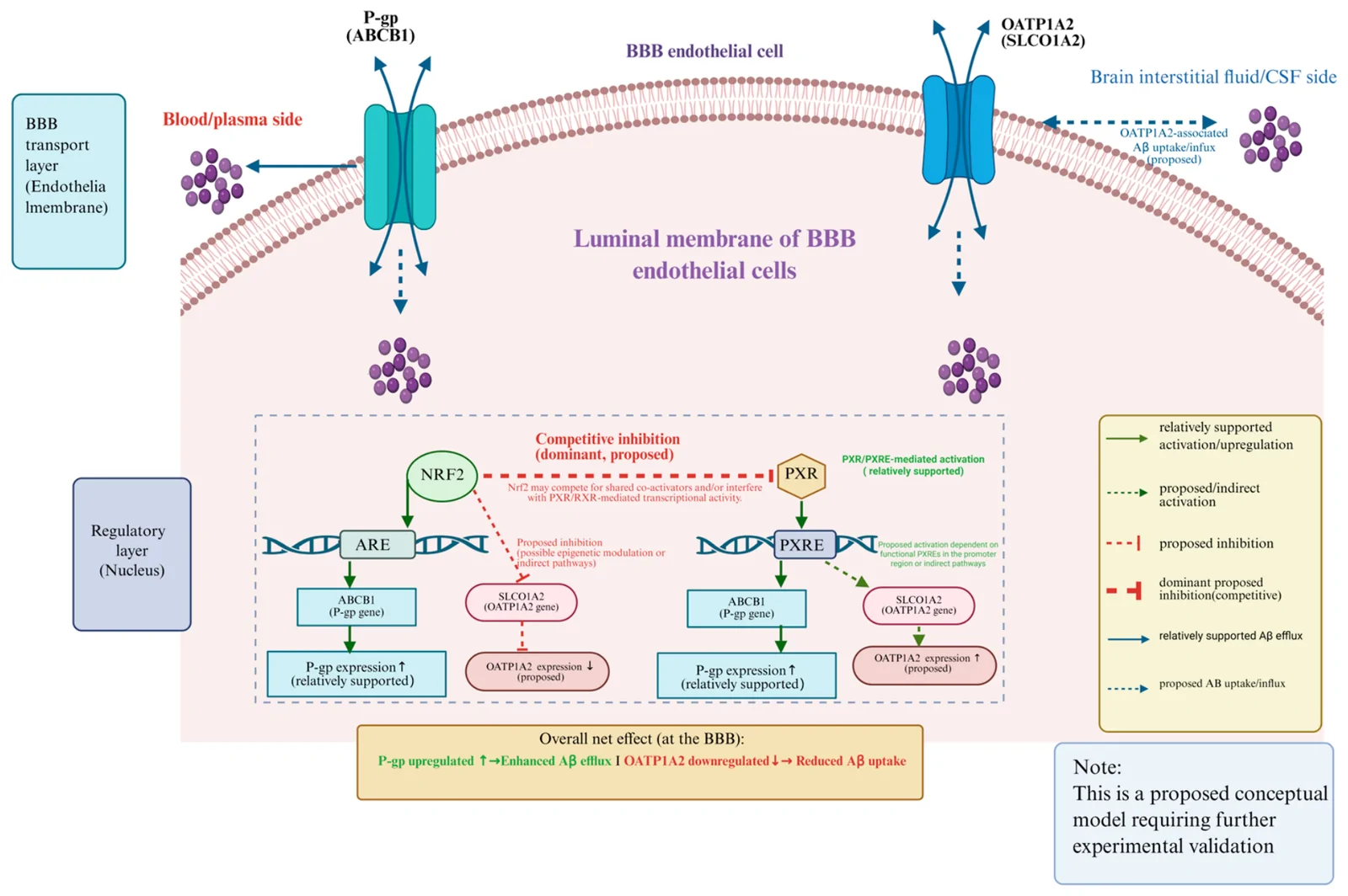
Integrated model of Nrf2–PXR crosstalk, OATP1A2/P-gp expression, and Aβ transport balance at the BBB. The schematic links a nuclear regulatory layer within the BBB endothelial cell with a membrane transport layer. Nrf2 may promote antioxidant response element (ARE)-associated *ABCB1*/P-gp expression and may indirectly influence *SLCO1A2*/OATP1A2 expression through proposed epigenetic or PXR-related mechanisms [21,22,23,27]. PXR, after interaction with RXR, may regulate PXR response element (PXRE)-associated transporter gene expression, including P-gp and OATP1A2 in context-dependent settings [25,27,28,29]. The proposed Nrf2–PXR/RXR crosstalk is conceptually supported by studies on transcriptional crosstalk, coactivator-dependent regulation, and retinoid X receptor alpha (RXRα)–Nrf2 interaction [30,31,32,33,34]. Functionally, this model proposes a possible shift toward increased P-gp-mediated Aβ efflux and potentially reduced OATP1A2-associated Aβ uptake [8,13,16,93]. Solid arrows denote relatively supported pathways, dashed arrows denote proposed or indirect pathways, and dashed red blunt-ended lines denote proposed inhibitory interactions. This model requires further validation in BBB-specific and AD-relevant experimental systems. Created in BioRender. Zhang, Y. (2026) https://BioRender.com/lmwgpqy.

Modulating BBB transport function remains a major challenge in AD research. As transcriptional regulators involved in oxidative stress responses, xenobiotic sensing, and transporter regulation, Nrf2 and PXR may influence BBB transporter homeostasis, particularly through regulatory effects on P-gp and potentially on OATP1A2 [21,26,27,28,29]. In parallel, OATP1A2/Oatp1a4-associated Aβ uptake and P-gp-mediated Aβ efflux may represent opposing components of the BBB Aβ transport balance [8,13,16,93]. However, the precise molecular mechanisms underlying Nrf2–PXR crosstalk at the BBB remain incompletely elucidated (Figure 3).

Current evidence suggests that Nrf2 activation may enhance ABC transporter expression and/or activity at blood–CNS barriers, whereas PXR signaling can regulate P-gp expression and transport function at the BBB [21,26,28,29]. At the same time, Nrf2–PXR/RXR-related interactions may be shaped by coactivator-dependent transcriptional crosstalk or RXRα–Nrf2 interactions [30,31,32,33,34]. Whether these interactions alter PXR ligand responsiveness, RXR heterodimerization, or PXR-mediated transcriptional regulation of OATP1A2 and P-gp in BBB endothelial cells remains unclear. Further studies are required to determine whether Nrf2 and PXR regulate OATP1A2 and P-gp independently, competitively, or cooperatively in BBB-specific and AD-relevant systems.

Based on the available evidence, a conceptual and testable “net-effect” model is proposed in this review, in which Nrf2–PXR crosstalk may shift BBB transport homeostasis toward enhanced P-gp-mediated Aβ efflux and potentially reduced OATP1A2-associated Aβ uptake. The P-gp-related component is supported by evidence linking Nrf2/PXR signaling to BBB P-gp regulation and by studies showing that P-gp contributes to Aβ clearance [8,21,26,28,29,93]. In contrast, the OATP1A2-related component remains more indirect, because OATP1A2-associated Aβ uptake and its regulation by Nrf2/PXR are mainly inferred from experimental cell models, non-BBB evidence, epigenetic regulatory mechanisms, hepatic Oatp studies, and promoter-level evidence [13,16,27,35,36,37]. Therefore, several key links in this model remain indirect or hypothetical, particularly the regulation of OATP1A2 by Nrf2 and PXR and the BBB-specific consequences of this regulation for Aβ influx. Further validation in human BBB models, AD-relevant experimental systems, and, where possible, human brain tissue is required. Clarification of this regulatory axis may deepen our understanding of BBB dysfunction in AD and guide future experimental investigations into BBB transporter regulation.

## Data Availability

No new data were created or analyzed in this study. Data sharing is not applicable to this article.

## Abbreviations

The following abbreviations are used in this manuscript:

ABC	ATP-binding cassette
AD	Alzheimer’s disease
Aβ	amyloid-β
APP/PS1	amyloid precursor protein/presenilin 1
ARE	antioxidant response element
BBB	blood–brain barrier
BCRP/ABCG2	breast cancer resistance protein/ATP-binding cassette subfamily G member 2
BMECs	brain microvascular endothelial cells
CBP	CREB-binding protein
CNS	central nervous system
CSF	cerebrospinal fluid
Cul3	Cullin 3
CYP	cytochrome P450
CYP2B6	cytochrome P450 family 2 subfamily B member 6
CYP3A4	cytochrome P450 family 3 subfamily A member 4
DBD	DNA-binding domain
DNMTs	DNA methyltransferases
GCN5	general control nonderepressible 5
GSH	glutathione
GSK-3β	Akt/glycogen synthase kinase 3 beta
H3K9Ac	histone H3 lysine 9 acetylation
HDAC6	histone deacetylase 6
HDACs	histone deacetylases
HO-1	heme oxygenase-1
K48	lysine 48
Keap1	Kelch-like ECH-associated protein 1
LBD	ligand-binding domain
LRP1	lipoprotein receptor-related protein 1
miRNA	microRNA
mRNA	messenger RNA
MRP2	multidrug resistance-associated protein 2
Neh4/5	Nrf2-ECH homology 4/5
Neh7	Nrf2-ECH homology 7
NQO1	NAD(P)H quinone oxidoreductase 1
NR1I	nuclear receptor subfamily 1 group I
Nrf2	Nuclear factor erythroid 2–related factor 2
OATP1A2	organic anion transporting polypeptide 1A2
p300	E1A-binding protein p300
PCAF	p300/CBP-associated factor
PET	Positron emission tomography
PI3K/Akt	phosphoinositide 3-kinase/protein kinase B
P-gp	P-glycoprotein
PXR	pregnane X receptor
PXRE	PXR response element
RAGE	receptor for advanced glycation end products
ROS	reactive oxygen species
RXR	retinoid X receptor
RXRα	retinoid X receptor alpha
SCE	Schisandra chinensis extract
SFN	sulforaphane
siRNA	small interfering RNA
SLCO	solute carrier organic anion transporter
SOD	superoxide dismutase
